# Application-Oriented Growth of a Molybdenum Disulfide (MoS_2_) Single Layer by Means of Parametrically Optimized Chemical Vapor Deposition

**DOI:** 10.3390/ma13122786

**Published:** 2020-06-20

**Authors:** Pinakapani Tummala, Alessio Lamperti, Mario Alia, Erika Kozma, Luca Giampaolo Nobili, Alessandro Molle

**Affiliations:** 1 IMM-CNR, Unit of Agrate Brianza, via C. Olivetti 2, I-20864 Agrate Brianza (MB), Italy; pinakapani.tummala@mdm.imm.cnr.it (P.T.); mario.alia@mdm.imm.cnr.it (M.A.); alessandro.molle@mdm.imm.cnr.it (A.M.); 2 CNR-SCITEC, via A. Corti 12, I-20133 Milano, Italy; erika.kozma@scitec.cnr.it; 3 Politecnico di Milano, Dipartimento di Chimica, Materiali e Ingegneria Chimica, Via Mancinelli 7, I-20131 Milano, Italy; luca.nobili@polimi.it

**Keywords:** 2D materials, transition metal dichalcogenides, molybdenum disulfide, chemical vapor deposition

## Abstract

In the 2D material framework, molybdenum disulfide (MoS_2_) was originally studied as an archetypical transition metal dichalcogenide (TMD) material. The controlled synthesis of large-area and high-crystalline MoS_2_ remains a challenge for distinct practical applications from electronics to electrocatalysis. Among the proposed methods, chemical vapor deposition (CVD) is a promising way for synthesizing high-quality MoS_2_ from isolated domains to a continuous film because of its high flexibility. Herein, we report on a systematic study of the effects of growth pressure, temperature, time, and vertical height between the molybdenum trioxide (MoO_3_) source and the substrate during the CVD process that influence the morphology, domain size, and uniformity of thickness with controlled parameters over a large scale. The substrate was pretreated with perylene-3,4,9,10-tetracarboxylic acid tetrapotassium salt (PTAS) seed molecule that promoted the layer growth of MoS_2_. Further, we characterized the as-grown MoS_2_ morphologies, layer quality, and physical properties by employing scanning electron microscopy (SEM), Raman spectroscopy, and photoluminescence (PL). Our experimental findings demonstrate the effectiveness and versatility of the CVD approach to synthesize MoS_2_ for various target applications.

## 1. Introduction

Recently, transition metal dichalcogenides (TMDs) are gaining tremendous interest for their potential use in wide range of applications [1]. Among TMDs, molybdenum disulfide (MoS_2_) has so far received the largest consideration for its promising potential in various fields such as electronics, optoelectronics, and electrocatalysis due to its unique optical, electrical, and chemical properties [2,3]. When thinned to a single layer, a strong intra-layer covalent-bonded MoS_2_ is obtained with a thickness of 0.7 nm [4], whereas in few-layer MoS_2_, the different layers are stacked together by weak van der Waals interlayer interactions [5]. As a TMD in the 2H structural phase, MoS_2_ undergoes a transition from an indirect bandgap (1.3 eV) in the bulk to a direct gap in the single layer (1.8 eV) [6]. Monolayer MoS_2_ has outstanding properties, such as wide direct bandgap, intense light–matter interactions, strong spin–orbit coupling, and high carrier mobility [2]. In electronics and optoelectronics, such characteristics make monolayer MoS_2_ particularly attractive for building field effect transistors, photodetectors, chemical sensors, and other thin transparent electronics with boosted performances [7,8,9]. Conversely, isolated MoS_2_ domains containing a high density of catalytic active edge sites are essential for applications in catalysis such as the hydrogen evolution reaction (HER) [10]. Clearly, the two types of characteristics mutually collide in the sense that the former one demands a superior structural quality aiming at a “perfect” material, while the latter one is closely related to defect engineering towards a tailored control of the imperfections. As such, it is demanding to develop structurally different standards of MoS_2_ nanosheets for a given application.

Traditionally, single and few-layer TMDs such as MoS_2_ can be obtained using mechanical exfoliation, chemical (solvent) exfoliation, or intercalation methods that are limited to research due to their uncontrolled and unscalable size, thickness, and non-uniformity. When considering synthesis approach, chemical vapor deposition (CVD) is an easy and cost-effective method for producing large-scale TMDs [11]. Attempts have been extensively reported from different research groups, who have already synthesized MoS_2_ films by CVD with the aim to get favorable layers for the fabrication of prototypical devices such as transistors, photodetectors, and HER cells [3,8,12]. Lee et al. reported an early strategy for growing large-area MoS_2_ atomic layers using a single hot-wall furnace [13,14]. Similar to this, Van der Zande et al. reported a clear route for synthesizing large MoS_2_ single-crystal domains from solid molybdenum trioxide (MoO_3_) and S precursors. They obtained highly crystalline monolayer MoS_2_ domains up to a lateral size of 123 µm [15]. However, synthesizing MoS_2_ with a controlled thickness over a large area with wafer scalability is still an open issue. To further push this nanomaterial in real-world applications, one should grow MoS_2_ with the desired thickness and high crystallinity over a large scale to address and target the specific requirements favoring the integration of MoS_2_ at the 2D scale in performing devices.

In this work, we report on a parametric study on the growth of MoS_2_ nanosheets under a chemical vapor phase reaction condition [16]. This condition is dictated by thermodynamic quantities and configurational details. The main parameters for the former quantities were the total and partial pressure, the substrate temperature, and the growth time, whereas the latter aspect was investigated by varying the substrate-to-source distance as a key parameter. Outcomes resulting from the parameter variability enabled us to identify the appropriate nanosheet for different target applications, such as transistors for nanoelectronics [17], photodetectors for optoelectronics [2,12], and energy storage devices [3], requiring specific characteristics. In detail, we systematically investigated the various parameters that influence the application-oriented MoS_2_ growth such as growth pressure, growth temperature, growth time, and MoO_3_ source-to-substrate position. Further, we described how to engineer the growth of a MoS_2_ monolayer by tuning the more relevant process parameters according to the desired film characteristics. By optimizing the growth conditions, we obtained a growth map for easily synthesizing MoS_2_ from a continuous monolayer to isolated domains. A set of characterization techniques, combining Raman spectroscopy, scanning electron microscopy (SEM), and photoluminescence (PL), was exploited to examine morphology, thickness (number of layers), crystal quality, and film structure. Our results make a strong roadmap for synthesizing large-area application-oriented MoS_2_ nanosheets using the CVD process.

## 2. Materials and Methods

### 2.1. Substrate Conditioning

The substrate was p-doped Si with a 90 nm thermally grown thick SiO_2_ layer on the growth side. The substrate was cleaved using a diamond-tipped scribe pen from a 4-inch wafer of SiO_2_/Si(100) to a 4 × 2 cm^2^ substrate. The cleaved substrate was cleaned with acetone and isopropanol for a few minutes to avoid any impurity on the surface, followed by drying using a stream of nitrogen gas. Subsequently, the cleaned substrate was immersed in a mixed solution of H_2_O_2_:H_2_SO_4_ (piranha solution) of volume ratio 1:3 for 2 h. This treatment in piranha solution cleans the surface from organic residues. Due to its strong oxidizing power, it helps to activate the SiO_2_ surface through hydroxylation, making it OH-terminated and, hence, highly hydrophilic. Perylene-3,4,9,10-tetracarboxylic acid tetrapotassium salt (PTAS) seeding promoter crystals (0.48 mg) were dissolved in 10 mL water. Using a micropipette, a small volume of PTAS solution (~10 µL) was spread on the clean substrate. Finally, the substrate was placed on a hot plate at 110 °C for 1 min to evaporate the water content from the solution on the surface.

### 2.2. Growth

**Figure 1:**
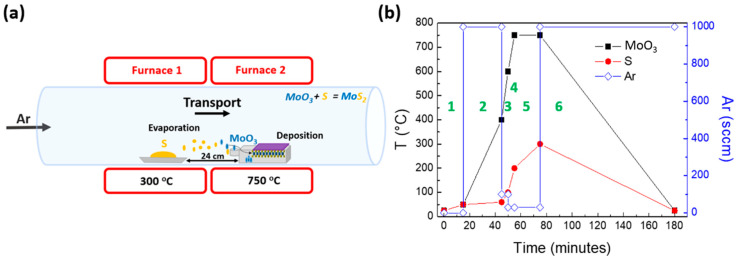
(**a**) Pictorial of the two-furnace chemical vapor deposition (CVD) apparatus for the growth of molybdenum disulfide (MoS_2_). (**b**) Furnace temperature profile as a function of process time (molybdenum trioxide (MoO_3_), black line; Sulphur (S), red line; left vertical axis) and argon (Ar) flux (blue line; right vertical axis) during the six steps of the growth process.

Molybdenum disulfide (MoS_2_) was grown on silicon substrates via CVD, from sulfur (S) (99.98%, Sigma-Aldrich, Darmstadt, Germany) and molybdenum trioxide (MoO_3_) (99.97%, Sigma-Aldrich, Darmstadt, Germany) powders. The amounts of S and MoO_3_ powders were 0.2 g and 0.001 g, respectively, as weighed by a microbalance. Subsequently, the measured quantities of S and MoO_3_ powders were placed in the center of ceramic or quartz crucibles. Our CVD system (Planartech UK Limited, Cambridge, UK) consisted of two separate thermal zones capable of providing precise control of the temperature for each precursor. In the CVD apparatus, the ceramic crucible containing the S precursor was placed in the center of the upstream furnace, while in the downstream heating zone the Si/SiO_2_ substrate was positioned face down on top of the quartz crucible containing the MoO_3_ powder. The MoS_2_ growth zone was located within the downstream heating zone, where the substrate was located. The distance between the two crucibles was kept fixed at 24 cm, as shown in Figure 1a. The temperature profile of the MoO_3_ (black line) and S (red line) precursors followed during the growth process is shown in Figure 1b, where the different process steps as a function of time are also presented. Figure 1b also shows the Ar flux (blue line) rate during the process, varying from 30 sccm to 1000 sccm in the different steps during the CVD process. The entire growth process developed under ambient pressure conditions.

### 2.3. Characterization

The as-grown MoS_2_ samples were characterized using Raman spectroscopy, scanning electron microscopy (SEM), photoluminescence (PL), atomic force microscopy (AFM), and X-ray photoelectron spectroscopy (XPS). Confocal Raman spectroscopy was performed using a Renishaw In-Via spectrometer (New Mills, Kingswood, Wotton-under-Edge, UK) equipped with a solid-state laser source of excitation wavelength 514 nm/2.41 eV in backscattering configuration. Particular care was put in the laser power, reduced down to 5% of the nominal power (i.e., below 1 mW range) to avoid sample damage. The morphology of the MoS_2_ layers was characterized using a Zeiss-SUPRA 40 field-emission SEM device (Oberkochen, Germany) in bright field mode. Complementary PL was acquired with the same Raman equipment by changing the instrument configuration; AFM topography was performed in tapping mode in a commercial Bruker Edge system (Billerica, MA, USA) using Si tips; and XPS was performed using a monochromated Al X-ray source (1486.6 eV) and a hemispherical analyzer in a PHI ESCA 5600 instrument (Chanhassen, MN, USA).

## 3. Results and Discussion

During the CVD synthesis, the growth of TMDs is governed by several factors, such as pressure, growth temperature, growing time, and substrate position. These factors are crucial for synthesizing high-quality and large-area monolayer MoS_2_ nanosheets. Hereafter, the role of each of these parameters on the lateral growth and morphology of MoS_2_ nanosheets is discussed in detail. Furthermore, the best growth conditions for obtaining large-area MoS_2_ nanosheets to isolated triangular domains are explained.

### 3.1. Pressure

**Figure 2:**
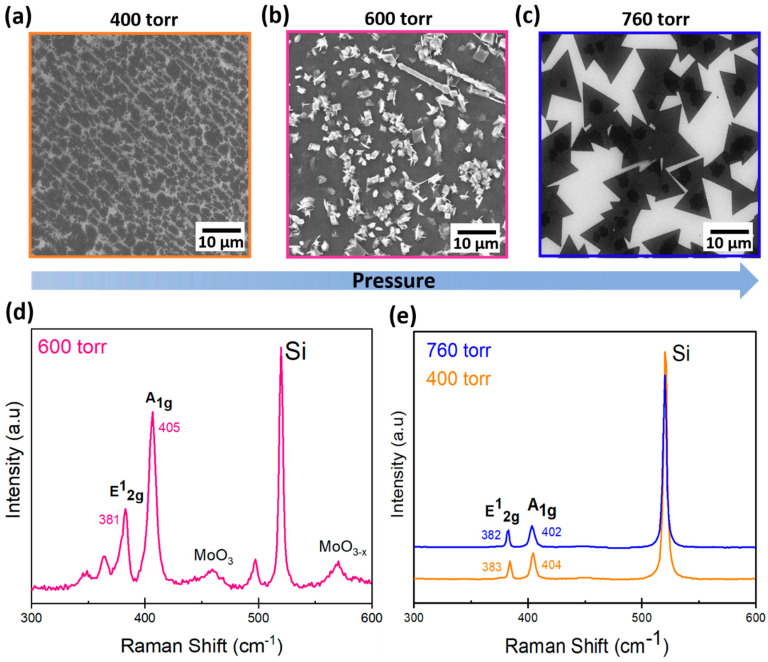
Scanning electron microscopy (SEM) images (planar view) of MoS_2_ grown on SiO_2_ at different pressures: (**a**) 400 torr, (**b**) 600 torr, and (**c**) 760 torr; (**d**) Raman spectrum of MoS_2_ at a pressure of 600 torr, and (**e**) Raman spectra of MoS_2_ on monolayer domains at 760 torr (blue) and on MoS_2_ particles at 400 torr (orange).

Keeping the growth temperature at 750 °C, we explored the effect of MoS_2_ growth at different pressures, namely 400 torr (5 × 10^4^ Pa), 600 torr (8 × 10^4^ Pa), and 760 torr (1 × 10^5^ Pa), varying the Ar flux rate from 30 sccm to 1000 sccm in multiple steps during the CVD process. At a process pressure of 400 torr, the growth results in the formation of grains that are clearly visible as dark regions in SEM images, as shown in Figure 2a. This effect is possibly due to the concentration gradient of the precursors over the substrate surface. Zheng et al. reported that such an effect occurs due to the large evaporation of MoO_3_ by lowering pressure [18]. As we see from the SEM image in Figure 2a and the Raman scattering spectra shown in Figure 2e, although the lateral coverage and the 2-layer thickness are good, the existence of randomly distributed MoS_2_ domains over the surface and wide grain boundaries still prevents us from setting the conditions for the desired polycrystalline MoS_2_ growth regime. Increasing the pressure up to 600 torr, the bulk growth is inhibited and the formation of mixed oxides Mo_x_S_y_O_z_ from the MoO_3_ and S precursors occurs, as revealed from the SEM analysis and Raman spectrum, as shown in Figure 2b,d. Surprisingly, 600 torr pressure remains a sharp regime for oxide deposition with bright square and wire-like structures as further confirmed from Raman characterization. At atmospheric pressure (760 torr), we observe the formation of well-defined triangular MoS_2_ domains with sharp edges, as shown in Figure 2c. Under such a condition, the amount of S in the gas phase is enough to promote a uniform and S-rich atmosphere in the reactor region where the substrate is placed so as to favor the chemical reaction between Mo and S. The presence of a S-rich atmosphere is confirmed by visual inspection of the quartz tube after the completion of the growth process, where residual solid S is found on the quartz tube walls. The number of layers at 600 torr was characterized by Raman spectroscopy, where a frequency difference between the Raman modes E^1^_2g_ (381) and A_1g_ (405) of 24 cm^−1^ allowed us to identify 4-layer thick MoS_2_ along with the presence of other peaks that are assigned to MoO_x_ phases, as shown in Figure 2d. Conversely, at 400 torr and 760 torr, the MoS_2_ particles and triangular domains, when characterized by Raman spectroscopy, revealed the main modes E^1^_2g_ (382 and 383) and A_1g_ (402 and 404) with a frequency difference of 20 cm^−1^ and 21 cm^−1^ (blue and orange line, respectively) corresponding to a thickness of 1 MoS_2_ layer (0.7 nm) and 2 layers, as shown in Figure 2e [19]. This condition is well described by a mass transport-limited regime where the growth is controlled by the amounts of precursors that are delivered, a condition that is typical of atmospheric pressure CVD [20]. In this regime, the control of the gas flow and the mean free path of reactants become particularly relevant [21]. Indeed, we confirmed that the atmospheric pressure CVD leads to a higher nucleation rate and a larger nucleation density compared to processes at lower pressures, thereby promoting the growth of a uniform monolayer MoS_2_ with a thickness of 0.7 nm at 760 torr. However, to enlarge the MoS_2_ growth over the substrate area and to ultimately obtain an extended continuous monolayer, other parameters apart from pressure control should be considered, as reported in the following sections.

### 3.2. Temperature

**Figure 3:**
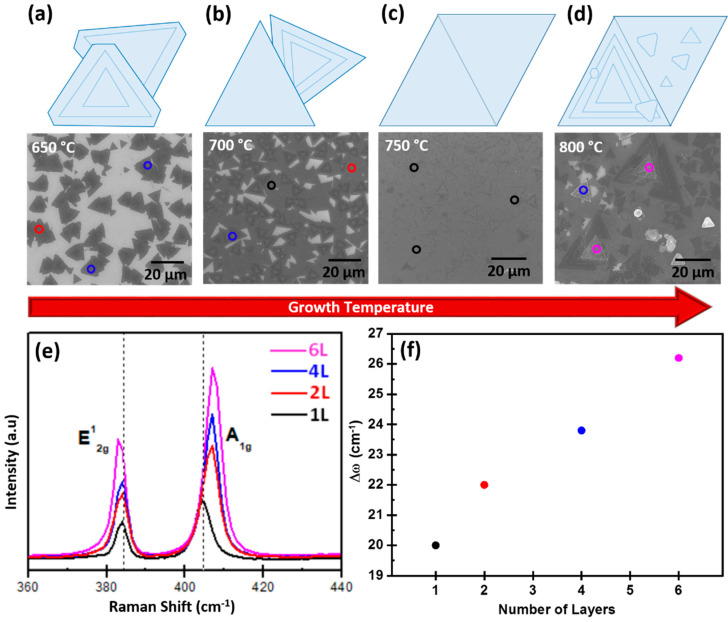
(Top) Schematic of MoS_2_ sheet orientation and (bottom) corresponding SEM images of synthesized MoS_2_ at various temperatures: (**a**) 650 °C, (**b**) 700 °C, (**c**) 750 °C, and (**d**) 800 °C. (**e**) Raman spectra at different growth temperatures with the corresponding number of layers. (**f**) Frequency difference between the A_1g_ and E^1^_2g_ modes as a function of the number of layers for spectra shown in panel (**e**).

Taking the ambient pressure (760 torr) as a fixed parameter, the MoS_2_ growth was investigated by changing the temperature from 650 °C to 800 °C within a 50 °C gradient range in step 5 of the CVD process in Figure 1b. Below 650 °C, MoS_2_ particles with no distinct morphology were observed, that are indicative of a poor growth environment. As the temperature was increased to 650 °C, the growth of isolated triangular domains was formed with truncated triangle-shaped crystals, as shown in Figure 3a. By raising the temperature to 700 °C, we observed that a high density of MoS_2_ domains increased with an exceptional coverage. Although there are many MoS_2_ domains with sharp edges, they appear as overlapped with adjacent bulk domains leading to a characteristic roughness regime, as shown in Figure 3b. Furthermore, when we raised the growth temperature to 750 °C, the increase in the lateral coverage and morphology with a uniform thickness of monolayer was clearly observed from Raman and SEM measurements (compared to Figure 3a,b,d). At this temperature, MoS_2_ domains with a large area coalesce so as to form a continuous monolayer, as shown in Figure 3c. Furthermore, increasing the temperature to 800 °C resulted in the formation of 3D islands on a continuous monolayer and small bilayer isolated triangular domains on the same MoS_2_ layer, as shown in Figure 3d. Similar to the parametric variation of pressure, the substrate temperature proves to have a strong impact on the thickness and morphology of grown MoS_2_. However, while pressure variations dictate the transition from a granular regime with a vertical island growth mode to a 2D layer regime, the variation of temperature at atmospheric pressure determines the condition of coalescence and the layer overgrowth on top of the interconnected triangular domain texture. In particular, our study proves that MoS_2_ coverage increases with the increasing growth temperature for a given value of pressure. In our case, we found that 750 °C was a favorable temperature to obtain a monolayer MoS_2_ growth regime with a high degree of coverage over a mm^2^ area. Further Raman measurements confirm the number of layers at different temperatures, as shown in Figure 3e. The frequency difference between the two Raman modes of A_1g_ and E^1^_2g_ identifies the layer number [19]. For the MoS_2_ films synthesized at 650 °C, 700 °C, 750 °C, and 800 °C, the number of MoS_2_ layers was revealed by measuring the Raman spectrum at different positions that are indicated by circular spots with different colors in Figure 3. The frequency differences between the Raman modes of the E^1^_2g_ and A_1g_ phonon modes were ~20 cm^−1^ (black), 22 cm^−1^ (red), 24 cm^−1^ (blue), and 26 cm^−1^ (pink) that corresponds to 1, 2, 4, and 6 layers, respectively. Therefore, by considering the three black circular spots from the Raman analysis at 750 °C, a uniform monolayer MoS_2_ growth is confirmed, as shown in Figure 3c. Conversely, in the other cases, there is a thickness variation of 1 to 6 layers that is confirmed from the Raman spectrum with different color spots at different positions, as shown in Figure 3e. In addition, the increase in the frequency difference between the A_1g_ and E^1^_2g_ phonon modes in the Raman spectra corresponding to the increase in the number of layers is indicated in Figure 3f.

### 3.3. Time (Thermal Budget)

**Figure 4:**
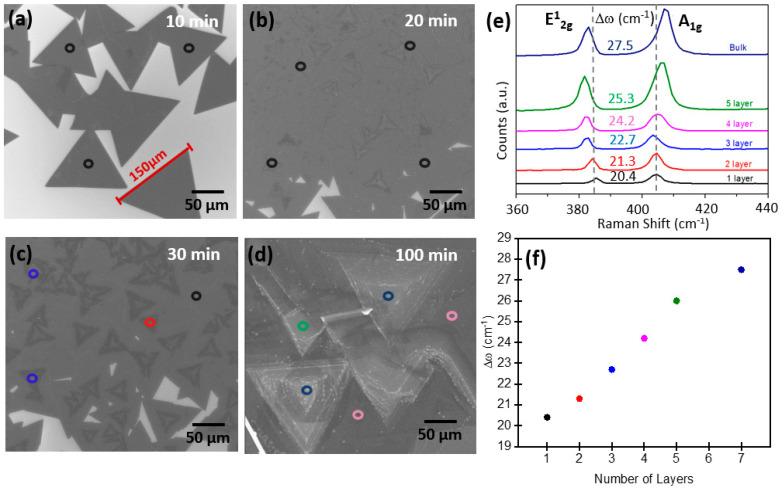
SEM images of obtained MoS_2_ growth at different growth times: (**a**) 10 min, (**b**) 20 min, (**c**) 30 min, and (**d**) 100 min. (**e**) Raman spectra taken at different positions as shown by circular spots in the SEM images with the corresponding frequency difference between the E^1^_2g_ and A_1g_ MoS_2_ phonon modes. (**f**) Frequency difference between the A_1g_ and E^1^_2g_ modes as a function of the number of layers.

We extended the study of MoS_2_ growth by exploring the role of the growth time at 760 torr (ambient pressure) and 750 °C. We explored the following growth times: 10 min, 20 min, 30 min, and 100 min at the given values of pressure and temperature. Referring to Figure 1b, by growth time we mean the duration of step 5 in the CVD process. Under the given condition, the growth time identifies the process thermal budget, namely the total amount of thermal energy transferred to the substrate during the process. The experimental results are shown in Figure 4a–e, which includes SEM images and Raman spectra. From the SEM images, it is evident that MoS_2_ morphology after growth varies from isolated large domains to a continuous layer growth by increasing the growth time. For instance, in the case of 10 min growth time, we strikingly found isolated monolayer domains as large as 150 µm in edge length and with sharp edges, as shown in Figure 4a. The uniform growth of the MoS_2_ layer is confirmed by measuring the Raman spectrum at different positions along the triangular domain area, here indicated by black circular spots. By considering the frequency difference between the E^1^_2g_ and A_1g_ phonon modes, a value of ~20.4 cm^−1^ is found indicating the presence of a monolayer, as shown in Figure 4e (black line) [22]. A slight shift (increase of ~0.4 cm^−1^) in the A_1g_ position in the Raman mode of single-layered MoS_2_ compared to the previously mentioned value of 20 cm^−1^ is due to the fact that the A_1g_ mode is strongly sensitive to electron doping. This is because the A_1g_ mode has a stronger electron–phonon coupling than the E^1^_2g_ mode [23]. Raising the growth time to 20 min, a tremendous increase in the lateral coverage with a continuous MoS_2_ monolayer is visible, as shown in Figure 4b. The growth time of 20 min is favorable for obtaining a uniform growth with large coverage up to an area of a few mm^2^. The above-mentioned conditions allowed us to get the best parametric conditions for uniform thickness and large-scale coverage MoS_2_ layer growth. However, if we extend the growth time to 30 min and above, additional layers start to grow from the center of the monolayer MoS_2_ domain, as is clearly shown in Figure 4c. Such layer generation at the center of the monolayer is also confirmed by the Raman spectrum in Figure 4e, where the frequency difference of 21.3 cm^−1^ (red line, 2 layers) and 22.7 cm^−1^ (blue line, 3 layers) is clearly enlarged with respect to the value of the monolayer, 20.4 cm^−1^ (black line, 1 layer). Considering a very long growth time (100 min), the MoS_2_ assumes the 3D island-like shape, typical of a bulk growth, as shown in Figure 4d. Such bulk growth is confirmed by the frequency difference of the Raman modes, which is ~27.5 cm^−1^ (Figure 4e, dark blue line) corresponding to a MoS_2_ film consisting of many layers, i.e., with bulk characteristics. Figure 4f shows a linearly increasing trend in the number of layers at an increasing value of the frequency difference between the A_1g_ and E^1^_2g_ phonon modes in the Raman spectra.

### 3.4. MoO_3_ Source to Substrate Relative Distance

Having systematically studied the effects of growth pressure, temperature, and time on the characteristics of the obtained MoS_2_ nanosheets, the source-to-substrate position during growth also plays an important role in achieving controllable thickness and lateral coverage.

**Figure 5:**
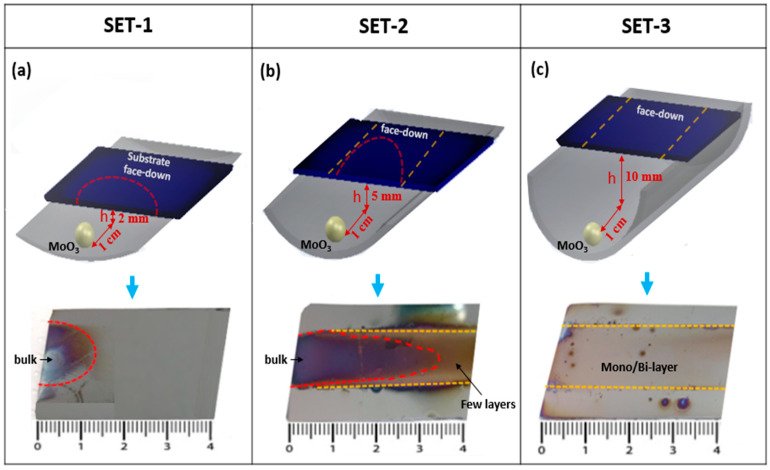
(Top) Schematic illustration of the vertical gap between the MoO_3_ source and the face-down substrate in the quartz crucible: (**a**) 2 mm (Set-1), (**b**) 5 mm (Set-2), and (**c**) 10 mm (Set-3) along with (Bottom) supporting images of MoS_2_ grown on Si/SiO_2_ substrates.

**Figure 6:**
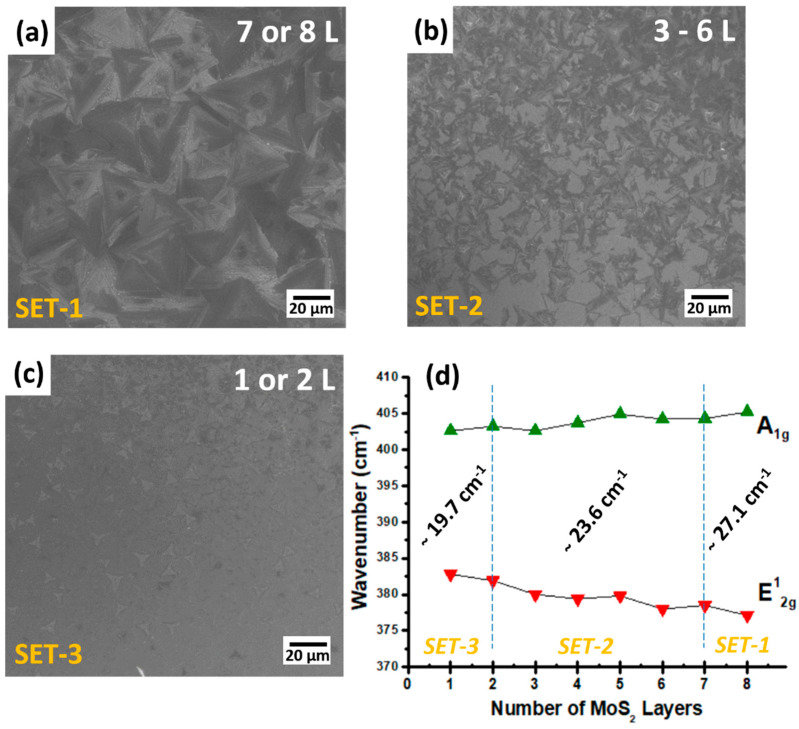
SEM images of MoS_2_ growth at different vertical gaps corresponding to three sets: (**a**) 2 mm (Set-1), (**b**) 5 mm (Set-2), and (**c**) 10 mm (Set-3). (**d**) Raman data assessment of the positions of the A_1g_ and E^1^_2g_ peaks depending upon the number of layers.

Here, we explored the growth employed under identical growth conditions in order to compare the growth pattern, number of layers, and flake size as a function of the relative vertical distance between the position of the MoO_3_ precursor and the substrate as illustrated in Figure 5a–c for the different conditions explored (set-1, -2, and -3). For this purpose, we used three quartz boats with different vertical heights, indicated by *h*, of 2, 5, and 10 mm. In set-1 (*h* = 2 mm), the deposited MoS_2_ nanosheets are very limited at the sample edge close to the MoO_3_ precursor, propagating up to 1 cm on the substrate surface, as shown by the red dashed region in Figure 5a. Such a MoS_2_ grown region consists of very thick and almost bulk MoS_2_ film, as observed by Raman spectra revealing a peak difference between the E^1^_2g_ and A_1g_ peaks of 27.1 cm^−1^, typical of bulk MoS_2_. Therefore, such a set condition is not effective to obtain 2D-layered growth of MoS_2_. In the case of set-2 (*h* = 5 mm), two types of MoS_2_ growth behavior can be readily discriminated, namely a plume-shaped one corresponding to a bulk deposition (red region) and a few layers all around extending up to the substrate edges where MoS_2_ reduces to a single layer, as indicated by the yellow dashed lines in Figure 5b. Raman investigation of the as-grown samples reveals a difference between the position of the E^1^_2g_ and A_1g_ peaks from 27.1 cm^−1^ down to 23.6 cm^−1^, typical of bulk to three layers of MoS_2_. In addition, in a few regions, a difference of ~20 cm^−1^ is detected, evidence of the presence of monolayer MoS_2_. It is worth noting that the region outside the red zone and within the delimiting yellow lines is the (large) region of the substrate where MoS_2_ is forming as domains of a few layers down to the monolayer. In the case of the set-3 configuration (*h* = 10 mm), we achieved mostly monolayer and bilayer MoS_2_ growth at the edges of the substrate, as indicated by the yellow dashed line. In addition, MoS_2_ domains with 3 or 4 layers are obtained at the center of the substrate at some minimal positions. In this configuration, the growth of 2D MoS_2_ is homogeneous and continuous compared to the previous cases with a reduced vertical gap. Raman spectroscopy investigation reveals a peak difference between the MoS_2_ main phonon modes (A_1g_, E^1^_2g_) in the range of 22 cm^−1^–19.7 cm^−1^, evidence of bilayer or monolayer MoS_2_. In this configuration, it is possible to obtain large-area crystalline and monolayer MoS_2_ domains. In addition, the as-grown MoS_2_ morphology and Raman analysis of the A_1g_ and E^1^_2g_ peak positions versus the number of MoS_2_ layers are shown in Figure 6a–d. Based on SEM and Raman data, MoS_2_ growth under set-1 conditions results in a bulk-like layer (7 or 8 layers). Similarly, in set-2 and set-3, the as-grown MoS_2_ films tend to multilayers (3–6 layers) and mono/bilayers (1 or 2 layers) as shown in Figure 6a–c. Figure 6d shows the peak position of the A_1g_ and E^1^_2g_ peak Raman modes as a function of the number of MoS_2_ layers. As the number of MoS_2_ layers increases, the frequency separation between the two modes tends to increase, as is clearly shown in Figure 6d. Therefore, we conclude that a 10 mm vertical gap is the best favorable condition to obtain uniformly continuous and large-area growth of single-layer and bilayer MoS_2_ [24].

### 3.5. CVD Growth of MoS_2_ Towards Target Applications

Because of the high requirement to produce and expand MoS_2_ from research to production level for its integration into various applications and to tackle this challenge, the CVD method shows a reliable way for producing large-scale, high-quality, thickness-controlled MoS_2_ nanosheets according to the target application. By tuning various factors like temperature, pressure, time, and vertical distance between the MoO_3_ source and the substrate, it is relatively easy to optimize the MoS_2_ growth. Herein, we demonstrated the easily tunable conditions that determine the high quality, thickness control, shape, and size from isolated MoS_2_ domains to continuous nanosheets suitable for integration into electronics, electrocatalysis, photonics (see the SEM and TEM images in Figures S1 and S2, Supplementary Materials, respectively). In general, monolayer MoS_2_ mainly addresses electronics and optoelectronic applications, because of its tunable bandgap with a semiconducting character or, with the limit of the monolayer, due to its direct bandgap transition, whereas 3D bulk to few-layer MoS_2_ growth is an essential platform for HER in electrocatalysis and supercapacitors, where surface-to-volume exposed active sites are a key parameter [25,26]. Since MoS_2_ is the most consistent potential candidate for many applications, it is essential to facilitate choosing among the growth conditions using fast and more cost-effective methods. Our characterization of MoS_2_ growth provides the parametric conditions that help to obtain the MoS_2_ morphologies that are specifically compliant with the two last requirements. Two different examples in this respect are discussed below for clarity.

#### 3.5.1. Electronics and Optoelectronics

There has been much interest in exploiting monolayer MoS_2_ nanosheets in field-effect transistors (FETs) and other potential devices. Monolayer MoS_2_ FETs offer some excellent electrical performance qualities in terms of good mobility, ON/OFF ratio, and high mechanical flexibility [1,12,27]. However, it is still difficult to obtain large-scale uniform MoS_2_ monolayers in order to fabricate more than a single FET device in the same integration process. For this scope, the bottom-up synthesis of atomically thin MoS_2_ using the CVD method was extensively demonstrated here. By selecting the process parameters for growth temperature at 750 °C, growth duration of 20 min, with a vertical height between the MoO_3_ source and substrate of *h* = 10 mm, and under an ambient pressure condition, it is possible to achieve the growth of highly polycrystalline monolayer MoS_2_ nanosheets on SiO_2_/Si substrate. Under such conditions, in the mass transport regime, the 3D-like growth mode of MoS_2_ growth is inhibited, possibly due to the minimization of the nucleation sites on the substrate; finally, MoS_2_ growth is extended and continuous, from monolayer up to the mesoscale. The vertical height is essential to allow the MoS_2_ film homogenous growth over the substrate surface, thus controlling the final thickness of the MoS_2_ layer. This easy method provides a high-quality MoS_2_ monolayer with lateral dimensions of a few millimeters with high growth yield, thus opening the opportunity to fabricate a greater number of FETs on a large area consisting of monolayer MoS_2_.

**Figure 7:**
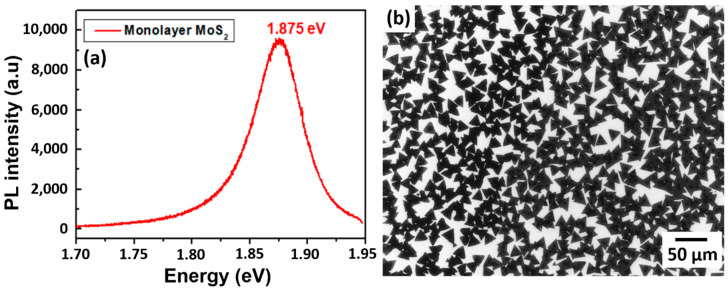
Characterization of monolayer MoS_2_ nanosheets: (**a**) photoluminescence (PL) spectrum showing a strong emission for a monolayer MoS_2_ and (**b**) SEM image of isolated highly dense MoS_2_ domains on SiO_2_/Si substrate.

For optoelectronic applications, photoluminescence (PL) spectroscopy investigation was done to verify the optical properties of the monolayer MoS_2_ nanosheet. The PL spectrum in Figure 7a indicates a PL response peaked around the optical bandgap of 1.875 eV on monolayer MoS_2_, which confirms the direct gap transition [28]. At room temperature, the pristine monolayer MoS_2_ shows a high-quality strong PL peak associated with the band-to-band optical transition at the K point. The direct optical transitions are expected to take place between the conduction band minimum and the two-valence band maxima at the K point of the Brillouin zone, which splits due to the spin–orbit coupling [29]. The obtained experimental bandgap values of strong emission for a monolayer MoS_2_ associated with A exciton at 1.875 eV is in agreement with the reported value [15]. The above-mentioned PL characterization confirms that the as-grown monolayer MoS_2_ nanosheets acquire an excellent optical response that is favorable in various optoelectronic applications. Furthermore, the topography monolayer MoS_2_ with a height profile of 0.7 nm thickness is confirmed by AFM measurement, as presented in Figure S3, Supplementary Materials. Therefore, the above-mentioned monolayer MoS_2_ nanosheets with different advantages help to push forward the applications of these TMD materials in the wide range of the optoelectronics field.

#### 3.5.2. Electrocatalysis (HER)

Highly dense MoS_2_ domains with active metal edge sites are evidence of catalytic activity. However, considerable efforts are still needed to grow MoS_2_ with a metallic 1T polymorph, which displays a more amenable charge conductivity throughout the layer [10]. Conversely, the mixed phase of 2H-1T MoS_2_ domains was also suggested as a satisfactory candidate for HER performance compared to 1T-MoS_2_ and 2H-MoS_2_ in terms of favorable free energy ΔG_H*_ [30]. To target HER utilizing the mixed phase of 2H-1T MoS_2_, we established a simple strategy to tune the 2H-1T MoS_2_ growth on SiO_2_/Si substrate. 2H-MoS_2_ with the presence of minor 1T observed from XPS characterization is shown in Figure S4, Supplementary Materials. The favorable CVD process parameters for MoS_2_ growth with a suitable morphology for electrocatalysis are a growth duration of 10 min at 700 °C, 760 torr, and a vertical height between the MoO_3_ source and substrate of *h* = 5 mm. Such conditions promoted highly polycrystalline MoS_2_ domains with sharp active edges. Growth temperature and time favor the formation of isolated MoS_2_ domains over a large area. In addition, the SEM morphological image of highly dense isolated as-grown MoS_2_ domains with exposed sharp edges is shown in Figure 7b. This above-mentioned MoS_2_ growth is much desired for accelerating the electrocatalysis process.

## 4. Conclusions

Through a systematic study of the growth pressure, temperature, time, and relative distance between MoO_3_ precursor and substrate, we were able to define a set of standardizing growth conditions to obtain MoS_2_ with a certain morphology and domain size, adapted for targeted applications. Based on the optimized experimental conditions, we successfully prepared large isolated triangular MoS_2_ domains and extended MoS_2_ growth size from several micrometers to a few millimeters. Our work confirms that the CVD approach is an easy and low-cost strategy for synthesizing MoS_2_ with improved crystalline quality, large area coverage, and uniform thickness down to the monolayer. This study also shows the ability to parametrically tune the growth mode from a uniform and large-scale coverage with well-defined PL signature to a laterally rough and isolated pattern of MoS_2_ domains in order to target optoelectronics and catalysis directions.

## Supplementary Materials

The following are available online at https://www.mdpi.com/1996-1944/13/12/2786/s1, Figure S1: SEM images of the triangular monolayer MoS_2_ domains and extended uniform monolayer growth obtained by means of the CVD growth. (a), (b) isolated MoS_2_ domains, (c), (d) extended continuos MoS_2_ growth, Figure S2: TEM cross-sectional image of bilayer MoS_2_ on SiO_2_/Si substrate, Figure S3: AFM image of a large monolayer triangular MoS_2_ domain (the black line in the AFM image indicates the height profile measurement shown exactly beside and the height profile collected at the step-edge of MoS_2_ and substrate). (a) AFM topography showing MoS_2_ domain in the area corresponding to the dashed square in the top right corner, where SEM of the whole MoS_2_ domain is shown, (b) height profile along MoS_2_ domain edge corresponding to the black line in panel (a), Figure S4: XPS spectrum for the binding energy of Mo and S of CVD-grown monolayer MoS_2_.
