# Supplementary material for: Application-Oriented Growth of a Molybdenum Disulfide (MoS2) Single Layer by Means of Parametrically Optimized Chemical Vapor Deposition
Source: Materials (Basel). 2020 Jun 20;13(12):2786. doi: 10.3390/ma13122786 (PMC7344844; doi:10.3390/ma13122786)
Supplement: Supplementary file 1 [file materials-13-02786-s001.pdf]

materials

Supplementary Materials

Application-Oriented Growth of Molybdenum Disulfide (MoS_2) Single Layer by Means of Parametrically Optimized Chemical Vapor Deposition

Pinakapani Tummala, Alessio Lamperti, Mario Alia, Erika Kozma, Luca Giampaolo Nobili and Alesssandro Molle

Scanning electron microscopy (SEM) and transmission electron microscopy (TEM)

The generality of our chemical vapor deposition (CVD) method in tuning and controlling the MoS_2 morphology from isolated MoS_2 domains to extended continuous MoS_2 layer growth is shown in SEM planar images in Figure S1 and in the cross-section TEM image in Figure S2, where the conformal and ordered growth of a MoS_2 bilayer is presented.

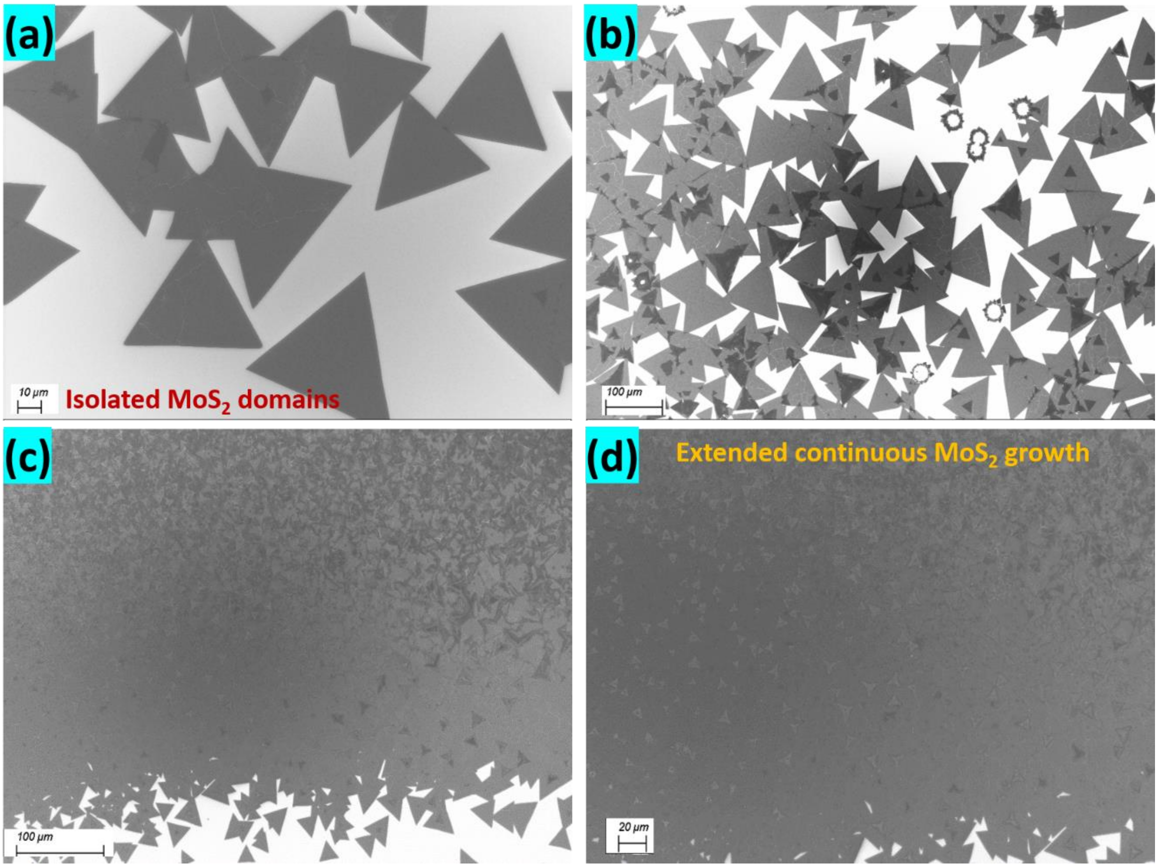

Figure S1. SEM images of the triangular monolayer MoS_2 domains and extended uniform monolayer growth obtained by means of the CVD growth. (**a**), (**b**) isolated MoS_2 domains, (**c**), (**d**) extended continuos MoS_2 growth.

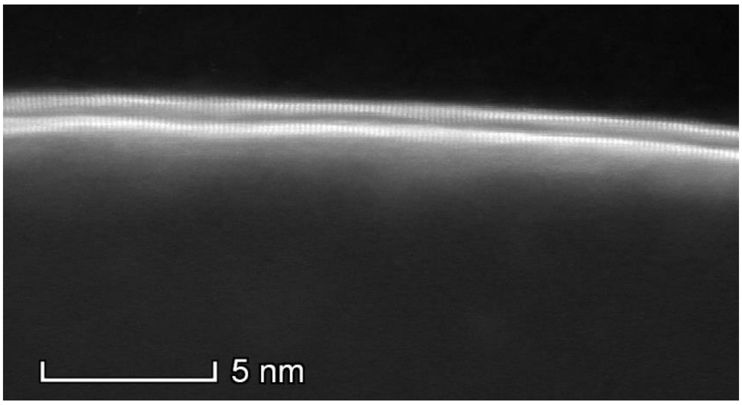

Figure S2. TEM cross-sectional image of bilayer MoS_2 on SiO_2/Si substrate.

Atomic force microscopy (AFM) and X-ray photoelectron spectroscopy (XPS)

To support and complement the characterization of our synthesized MoS_2 by CVD, we employed AFM topography to detail the characteristics of the MoS_2 monolayers. Figure S3 shows in detail a triangular MoS_2 monolayer domain with sharp edges. The height profile collected at the step-edge between the MoS_2 film and substrate indicated with the solid line (black) and shown in Figure S3 reveals a thickness of ~0.7 nm, which is consistent with the value obtained from the exfoliated MoS_2 monolayer. Because of this, and considering the TEM analysis in Figure S2, the large triangular MoS_2 domains are of high quality, without the presence of any defect between the MoS_2 domain and the SiO_2 substrate surface.

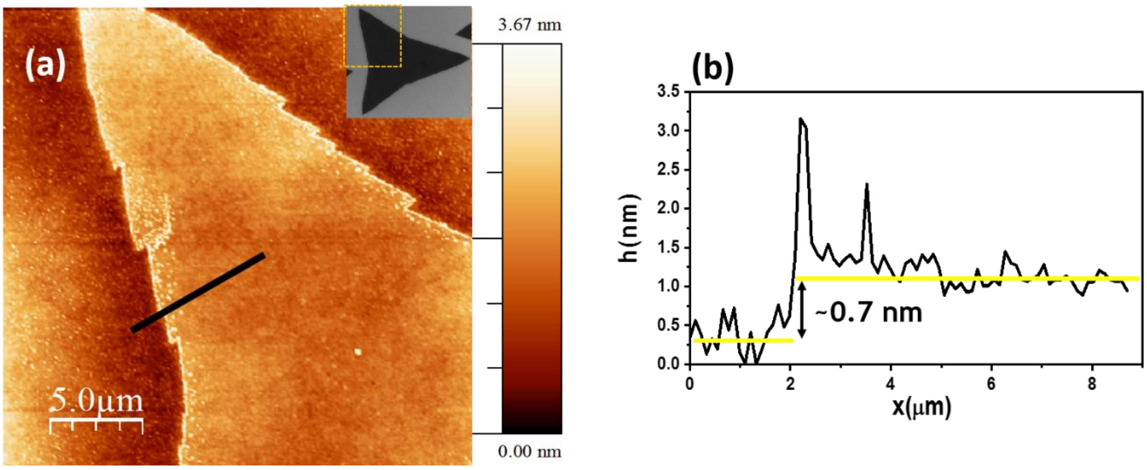

Figure S3. AFM image of a large monolayer triangular MoS_2 domain (the black line in the AFM image indicates the height profile measurement shown exactly beside and the height profile collected at the step-edge of MoS_2 and substrate). (**a**) AFM topography showing MoS_2 domain in the area corresponding to the dashed square in the top right corner, where SEM of the whole MoS_2 domain is shown, (**b**) height profile along MoS_2 domain edge corresponding to the black line in panel (**a**).

The elemental composition and chemical bonding of the grown MoS_2 nanosheets have been checked by XPS measurements. XPS data were fitted with pseudo-Voigt functions for the deconvolution of the peaks and doublet components considering a spin–orbit splitting of 3.1 eV for the 3d core levels of Mo, as shown in Figure S4. We found that the Mo 3d core-level line has Mo $3d_{5/2}$ at 230.6 eV and Mo $3d_{3/2}$ at 233.7 eV (green line), while the shoulder peak related to S 2s is at 227.8 (red line). The peak positions are indicative of a MoS_2 arranged in a majority trigonal prismatic 2H phase. Furthermore, an additional Mo 3d component observed at a binding energy of Mo $3d_{5/2}$ at 235 eV and Mo $3d_{3/2}$ at 238.1 eV (blue line) is related to a minor amount of an octahedral phase.

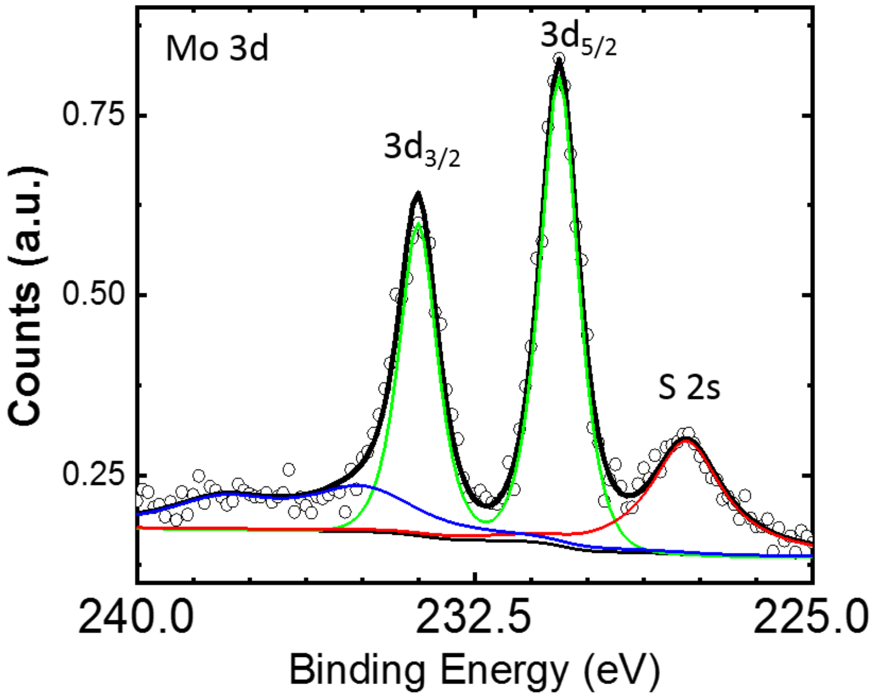

Figure S4. XPS spectrum for the binding energy of Mo and S of CVD-grown monolayer MoS_2.
